# Untargeted Urinary Proteomics Uncovers Nephroprotective and Systemic Adaptations after Obesity Surgery-Induced Weight Loss

**DOI:** 10.1021/acs.jproteome.5c00500

**Published:** 2025-12-10

**Authors:** Pedro R. Pereira, David F. Carrageta, Bárbara Guerra-Carvalho, Patrícia C. Braga, João Pereira, Sofia S. Pereira, Mário Nora, Marta Guimarães, Anabela Rodrigues, Mariana P. Monteiro

**Affiliations:** † Department of Nephrology, Unidade Local de Saúde de Santo António (ULS Santo António) - Porto, Largo Professor Abel Salazar, 4099-001 Porto, Portugal; ‡ Unit for Multidisciplinary Research in Biomedicine (UMIB), School of Medicine and Biomedical Sciences (ICBAS), 26706 University of Porto, Rua de Jorge Viterbo Ferreira n.° 228, 4050-313 Porto, Portugal; § ITR - Laboratory for Integrative and Translational Research in Population Health, Rua das Taipas 135, 4050-600 Porto, Portugal; ⊥ LAQV-REQUIMTE, Department of Chemistry, University of Aveiro, Campus Universitário de Santiago, 3810-193 Aveiro, Portugal; # General Surgery Department and CRI for the surgical Treatment of Obesity and Metabolic Diseases, ULS Entre o Douro e Vouga, Rua Dr. Cândido de Pinho, 4520-221 Santa Maria da Feira, Aveiro, Portugal

**Keywords:** obesity, obesity surgery, bariatric interventions, urine proteomics, mass spectrometry, obesity-related kidney disease

## Abstract

Weight loss induced by bariatric surgery (BS) has a profound impact on several biological systems. This study aimed to identify urinary proteins reflecting kidney and systemic adaptations to weight loss in patients with obesity before and after BS. Urine samples from individuals with obesity (*n* = 16) were collected before and two years after BS. Untargeted high-resolution LC-MS with label-free quantification was used to assess urinary proteome changes. Among the 2347 identified proteins, 1016 depicted a significantly different abundance postsurgery (*p* < 0.05). In particular, 54 proteins were either upregulated (*n* = 42) or downregulated (*n* = 12) by at least 50% (≥1.5-fold). Protein functional classification revealed associations with immune function (*n* = 17; e.g., protein S100-A9, α-1-acid glycoproteins); cytoskeleton/cell adhesion (*n* = 11; e.g., supervillin, ezrin, periplakin), and kidney adaptation (*n* = 11; e.g., elongation factor 1-α 1, megalin, cubilin). A decrease in inflammation protein markers (α-1-acid glycoproteins), alongside an increase in proteins associated with immune modulation and oxidative stress protection (dipeptidase 1, heat shock cognate 71 kDa protein) were observed. Overall, the urinary proteome suggests changes in inflammation and oxidative stress status, as well as in kidney function and cellular organization succeeding BS. Our results reveal potential novel pathways contributing to systemic modifications and nephroprotective effects of BS-induced weight loss.

## Graphical abstract

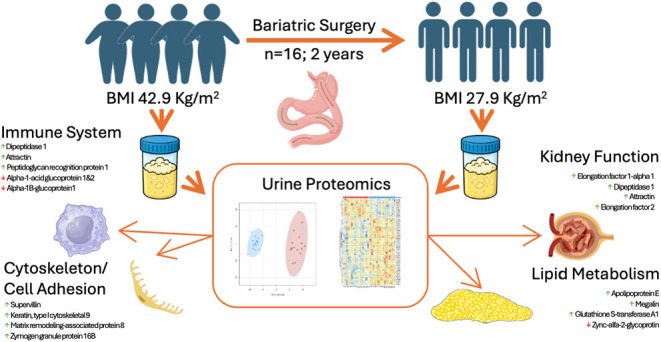

## Introduction

Obesity is characterized by an excessive accumulation of adiposity to the extent of impairing normal biological functions. Individuals with obesity often present a chronic low-grade inflammatory state and profound metabolic alterations that are associated with insulin resistance and an increased risk of diabetes, hypertension, and dyslipidemia.^1^ Obesity also has profound effects on kidney health, being an independent risk factor for kidney disease besides contributing to kidney disease progression, irrespective of the underlying etiology.^2^ Obesity is associated with a secondary form of focal segmental glomerulosclerosis termed obesity-related glomerulopathy (ORG), in which up to one-third of affected individuals progress to end-stage kidney disease.^3^ Slowly progressive proteinuria is the most common clinical presentation of ORG; however, histologic alterations linked to excess adiposity are often present long before clinical manifestations become evident.^4,5^ The mechanisms by which obesity promotes kidney injury remain incompletely understood, although several hypotheses have been proposed as contributors, including glomerular hyperfiltration, activation of the renin-angiotensin-aldosterone system, ectopic lipid accumulation, and exposure to circulating adipokines.^6^

Effective and sustained weight loss achieved through surgical treatment for obesity, so-called bariatric surgery (BS), was demonstrated to counteract the risk of developing obesity-related disorders, as well as to improve or even reverse previously established obesity-related health conditions.^7^ Weight loss has been shown to have beneficial effects on obesity-related kidney disease, potentially reducing urinary protein loss and improving kidney function or slowing disease progression.^8^ In patients submitted to bariatric surgery, studies have shown significant reductions in proteinuria or even normalization, as well as of glomerular filtration rate (GFR) and kidney plasma flow in patients with glomerular hyperfiltration.^9–13^

Proteome analysis offers a precise depiction of cellular and tissue functional dynamics under both normal and pathological conditions. Therefore, proteomics is a powerful tool for identifying potential biomarkers that reflect cellular processes, holding the promise of detecting protein expression changes that may signal physiological or pathological shifts.^14^

Urine is a widely available biofluid whose collection can be noninvasive, making it a valuable resource for biomarker discovery and diagnostic purposes.^15^ In recent years, numerous studies have investigated proteomic changes in various conditions, including cardiovascular disease,^16^ diabetes,^15,17^ and chronic kidney disease.^18,19^

Previous studies have focused on proteomic changes in the setting of obesity, although these studies were performed in biological samples other than urine, such as blood, adipose tissue, muscle, and reproductive system cells.^20^ These studies revealed an altered relative abundance of proteins involved in metabolic pathways, oxidative stress responses, inflammatory processes, protein folding, coagulation, and the structure/cytoskeleton. For instance, there is a large volume of accumulated evidence that weight loss following BS leads to adaptations of immune cell populations and allows improvements in inflammatory markers.^21,22^

Although previous studies have explored urinary proteomic alterations in obesity and following bariatric surgery, few have employed a longitudinal design with paired pre- and postsurgical samples and stringent exclusion criteria, thus limiting the reach of the conclusions.^23^

In this study, we aimed to characterize the urine proteome before and after BS to identify proteins potentially associated with kidney and systemic adaptations to weight loss.

## Experimental Procedures

### Study Population

This study included patients with obesity referred for BS at a single public bariatric center in 2020. Eligibility for BS was determined by a body mass index (BMI) > 40 kg/m^2^ or a BMI > 35 kg/m^2^ with obesity-related comorbidities.^24^ Exclusion criteria included the presence of diabetes mellitus (HbA1c > 6.5% or treatment with antidiabetic drugs regardless of HbA1c value), prediabetes under metformin treatment (HbA1c between 5.7 and 6.5%), neoplastic diseases, chronic inflammatory disorders, hypercortisolism, or overt kidney function alterations defined by a reduction in estimated glomerular filtration rate (eGFR) or confirmed proteinuria >300 mg/day. Patients with low 24-h urine collection volumes (<800 mL) were also excluded, as this volume was considered insufficient to ensure accurate creatinine clearance and proteinuria measurements. All patients enrolled in the study provided written informed consent to participate. The study protocol received prior approval from the Institutional Review Board (CA-014/20-Ot_MP/CC).

### Data Acquisition

Comprehensive demographic, clinical, and biochemical pre- and postsurgical (2-year) data were collected for each participant, as depicted in Table 1. Additional assessments included urinalysis, albuminuria, and proteinuria measurements from 24-h urine collections. The 2021 CKD-EPI Creatinine equation was used to estimate the glomerular filtration rate (GFR).^25^ Patients were instructed to perform 24 h urine collections on two consecutive days prior to blood sampling. Both oral and written instructions were provided to ensure a proper collection technique. Urinary albumin, protein, creatinine, and cortisol concentrations were measured from 24 h urine collections. The cohort was prospectively followed for a period of 2 years.

**Table 1:** Pre- and Postoperative Clinical and Biochemical Data of the Participants Included in This Study^a^,^b^,^c^

**Clinical and Biochemical Parameters**	**Presurgery**	**Postsurgery**	**Reference ranges**	***p*-value**
Weight (kg)	112.2 ± 19.7	72.9 ± 12.6		<0.001
BMI (kg/m^2^)	42.9 ± 5.6	27.9 ± 3.5		<0.001
Hypertension (*n*, %)	4 (25.0%)	1 (0.6%)		0.161
Dyslipidemia (*n*, %)	4 (25.0%)	0 (0%)		0.051
ARBs/ACEi (*n*, %)	4 (25.0%)	1 (0.6%)		0.161
Statin (*n*, %)	2 (12.5)	0 (0%)		0.242
Hemoglobin (g/dL)	13.8 ± 1.6	13.3 ± 1.1	12.0–16.0	0.120
Creatinine (mg/dL)	0.8 ± 0.1	0.7 ± 0.1	0.6–1.1	<0.001
Urea (mg/dL)	31.0 ± 7.0	29.6 ± 6.8	21–43	0.388
CKD-EPI (mL/min/1.73m^2^)	100.5 ± 12.9	108 ± 12.8	>90	0.025
Uric Acid (mg/dL)	5.5 ± 1.3	4.8 ± 0.8	2.6–6.0	0.031
Glycated Hemoglobin (%)	5.3 ± 0.4	#	4.3–6.1	
Total Cholesterol (mg/dL)	196.0 ± 36.0	166.4 ± 23.9	<200	0.005
HDL Cholesterol (mg/dL)	49.0 ± 10.0	54.3 ± 11.0	>55	0.475
LDL Cholesterol (mg/dL)	140.0 ± 37.0	97.2 ± 27.8	<100	0.002
Triglycerides (mg/dL)	110.0 ± 66.0	74.7 ± 28.5	<150	0.013
Total Serum Proteins (g/dL)	7.1 ± 0.4	6.7 ± 0.4	6.4–8.3	0.001
Albuminuria (mg/24h)	14.8 ± 12.2	#	<30	
Proteinuria (mg/24h)	130.1 ± 79.7	#	<150	
Creatinine Clearance (mL/min)	138.6 ± 25.8	#	90–130	
Urinary Cortisol (nmol/24h)	131.6 ± 48.3	#	11.8–485.6	

a Note: # Only assessed before surgery to evaluate exclusion criteria.

b Values are presented as mean ± standard deviation (SD) for continuous variables and *n* (%) for categorical variables.

c Legends: BMI: body mass index; ARBs: angiotensin II receptor blockers; ACEi: angiotensin-converting enzyme inhibitors; CKD-EPI: chronic kidney disease epidemiology collaboration (estimated glomerular filtration rate); HDL: high-density lipoprotein; LDL: low-density lipoprotein.

### Proteomic Sample Preparation and Analysis

Urine samples for proteomic analysis were lyophilized and processed following the solid-phase-enhanced sample preparation (SP3) protocol.^26,27^ Proteins were enzymatically digested using trypsin/LysC as previously described.^26^ Protein identification and quantification were performed using a nanoLC-MS/MS equipped with a Field Asymmetric Ion Mobility Spectrometry (FAIMS) interface. The system comprised a Vanquish Neo liquid chromatography system coupled to an Eclipse Tribrid Quadrupole-Orbitrap-Ion Trap mass spectrometer (Thermo Scientific, San Jose, CA). Each sample (250 ng of peptides) was loaded onto a trapping cartridge (PepMap Neo C18, 300 μm × 5 mm inner diameter, Thermo Scientific, Bremen, Germany). The trap column was then switched in-line to a μPAC Neo 50 cm column (COL-nano050NeoB) coupled to an EASY-Spray nano flow emitter (10 μm i.d., Thermo Scientific, Bremen, Germany). Peptide separation was performed over 130 min using a gradient of solvent A (0.1% formic acid) and solvent B (80% acetonitrile, 0.1% formic acid) at a flow rate of 750 nL/min: 0.1 min (1% B to 4% B), 1.9 min (4% B to 7% B), followed by a flow reduction to 250 nL/min with subsequent gradients: 0.1 min (7.0 to 7.1% B), 80 min (7.1% B to 22.5% B), 30 min (22.5% B to 40% B), 8 min (40% B to 99% B), and 9.9 min at 99% B. The column was then equilibrated with 1% B. Data acquisition was controlled using Xcalibur 4.6 and Tune 4.0.4091 software (Thermo Fisher Scientific, Waltham, MA, USA). Mass spectrometry (MS) was performed in Data-Dependent Acquisition (DDA) mode with the Orbitrap detector at a resolution of 120,000 in positive mode, quadrupole isolation, scan range of *m*/*z* 375–1500, RF Lens 30%, standard AGC target, and automatic maximum injection time. FAIMS mode was set with standard resolution, total carrier gas flow at 4 L/min, and compensation voltages of −45, −60, and −75 V (cycle time: 1 s). Internal mass calibration was performed using Run-Start Easy-IC. Filtering conditions included MIPS, monoisotopic peak determination set to peptide, charge state range of 2–7, dynamic exclusion of 30 s, and an intensity threshold of 5.0e3. MS/MS acquisition was performed using quadrupole isolation with a 1.8 *m*/*z* window, higher-energy collisional dissociation (HCD) at 30% collision energy, ion trap detection at a rapid scan rate, automatic scan range mode, a normalized AGC target of 100%, and a maximum injection time of 35 ms. Data were acquired in centroid mode.

### Data Processing and Quantification

Raw MS data were processed using Proteome Discoverer 3.1.0.638 software (Thermo Fisher Scientific, Waltham, MA, USA) and searched against the UniProt *Homo sapiens* reviewed proteome database (2024_01, containing 20,418 entries). A common contaminant protein list from MaxQuant was also included.^28^ Peptide identification was performed by using MSPepSearch and Sequest HT search engines. Ion mass tolerances were set to 10 ppm for precursor ions and 0.5 Da for fragment ions, with a maximum allowance of two missed cleavage sites. Cysteine carbamidomethylation was set as a fixed modification, while methionine oxidation, glutamine and asparagine deamidation, peptide-terminal glutamine to pyroglutamate conversion, and protein N-terminal modifications (acetylation, methionine loss, and methionine loss plus acetylation) were defined as variable modifications. Peptide confidence was set to high, with validation performed using the Percolator algorithm (maximum delta Cn: 0.05; target false discovery rate (FDR), strict: 0.01; relaxed: 0.05; validation based on q-value). Protein quantification was performed using the Minora feature detector in the processing step. Precursor ion quantification was carried out at the consensus step using unique plus razor peptides, intensity-based precursor abundance, and normalization based on the total peptide amount. The mass spectrometry proteomics data have been deposited to the ProteomeXchange Consortium via the PRIDE^29^ partner repository with the data set identifier PXD063904 and 10.6019/PXD063904. Raw data containing all of the identified and quantified proteins can be found in Supporting Table 1.

### Experimental Design and Statistical Rationale

Pre- and postoperative clinical and biochemical data are expressed as mean ± standard deviation (SD), unless otherwise stated. The Shapiro-Wilk test was employed to assess the normality of the data distribution. For the comparison of the multiple variables pre- and postsurgery, a paired analysis was conducted using a paired two-tailed *t*-test or a Wilcoxon test, depending on the normality of the data. Nominal variables were analyzed using the χ^2^ test. These statistical analyses were performed using IBM SPSS (version 28.0) for IOS.

Only proteins with a minimum number of two unique peptides and high confidence were considered for the proteome data analysis. Proteins with low availability (>50% of missing values) and classified as contaminants were removed from the analysis. Proteomics data were normalized by total intensity, log-transformed, and EigenMS normalized using R version 4.3.3 in RStudio version 2023.12.1 (Posit PCB, Boston, MA, USA).^30^ Missing values were replaced by LoDs (1/5 of the minimum positive value of each variable). Statistical analysis was performed in Metaboanalyst 6.0 (https://metaboanalyst.ca/).^31^ Initial data structure was evaluated using principal component analysis (PCA), and a PERMANOVA test was used to assess potential centroid differences. A paired two-tailed *t*-test was used to compare each protein relative abundance between groups with False Discovery Rate (FDR) applied as a *posthoc* correction. Hierarchical clustering heatmap was created from autoscaled data using the pheatmap R package (clustering of features was performed as follows: clustering distance: “Euclidean”; clustering method: “ward.D”). All of the other plots were created using GraphPad Prism 9.5 software (GraphPad, Boston, MA, USA). A *p* < 0.05 was considered statistically significant.

## Results

### Patients’ Characteristics

The pre- and postsurgical demographic, clinical, and biochemical data of the 16 patients included in the study are shown in Table 1. The cohort had a mean age of 44.0 ± 12.0 years before surgery, and 75.0% (*n* = 12) of the participants were females. Patients underwent different types of BS procedures, namely, Roux-en-Y gastric bypass (RYGB) (*n* = 11; 68.8%); single anastomosis duodenal-ileal bypass with sleeve gastrectomy (SADIS-S) (n = 4; 25.0%); and gastric sleeve (*n* = 1; 6.2%). Before BS, 4 patients had hypertension (25.0%), and 3 (18.8%) had dyslipidemia, but 2 years after BS, only one patient had persistent hypertension (*n* = 1; 6.2%), and none had dyslipidemia. Interventions were very effective at achieving significant weight loss, representing an average percentage of excess BMI loss (%EBMIL) of 85.4 ± 21.8% and an average percentage of total weight loss (%TWL) of 35.7 ± 9.4%, at 2 years after surgery. At baseline, patients did not depict alterations of the kidney function parameters besides a creatinine clearance above the upper limit, and, although there were statistically significant differences pre- and postsurgery in CKD-EPI values, these are not considered clinically relevant as both fell within the normal reference range.

### Individuals with Obesity Display a Distinct Urinary Proteome after Weight Loss

A total of 2347 proteins were identified in the urine samples, with 1016 showing significantly different expression (*p* < 0.05) when pre- and postsurgery proteomic profiles were compared (Figure 1). A complete list of the analyzed (Supporting Table 2) and altered proteins (Supporting Table 3) can be found in the Supporting Information. The Principal Component Analysis (PCA) demonstrated a clear separation between pre- and postsurgery urine proteomic profiles, with nonoverlapping 95% confidence intervals (Figure 2). A PERMANOVA test (*F* = 49.54; *R* ^2^ = 0.6228; *p* = 0.0010) confirmed significant differences in urine proteome composition, indicating a systematic shift in protein expression patterns postsurgery. PCA analysis stratified by sex (Supporting Figure 1) can be found in the Supporting Information.

**Figure 1:**
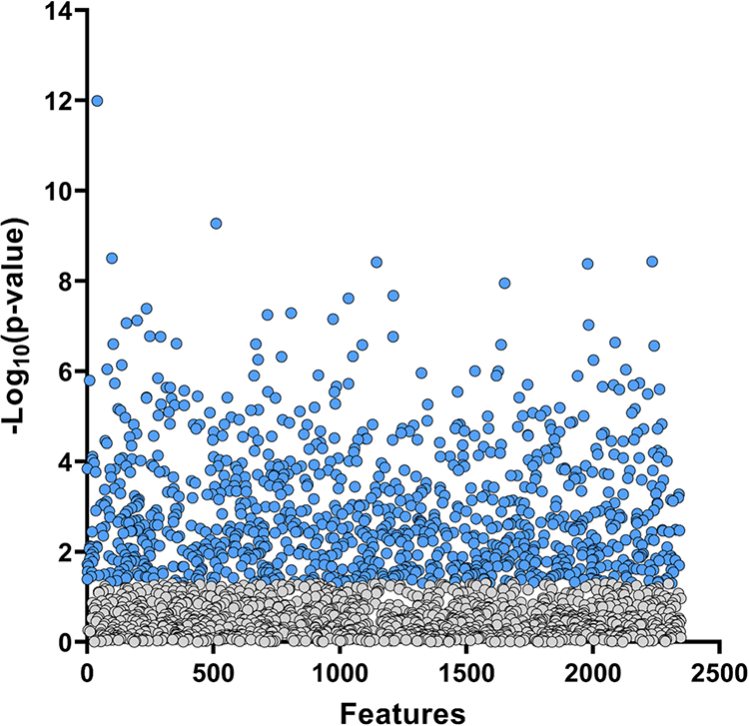
Urinary protein relative abundance in samples of individuals with obesity pre- and postbariatric surgery. Each point corresponds to a detected protein, with blue dots indicating proteins that are differentially expressed (*p* < 0.05). From the 2347 proteins identified, 1016 were significantly different between groups.

**Figure 2:**
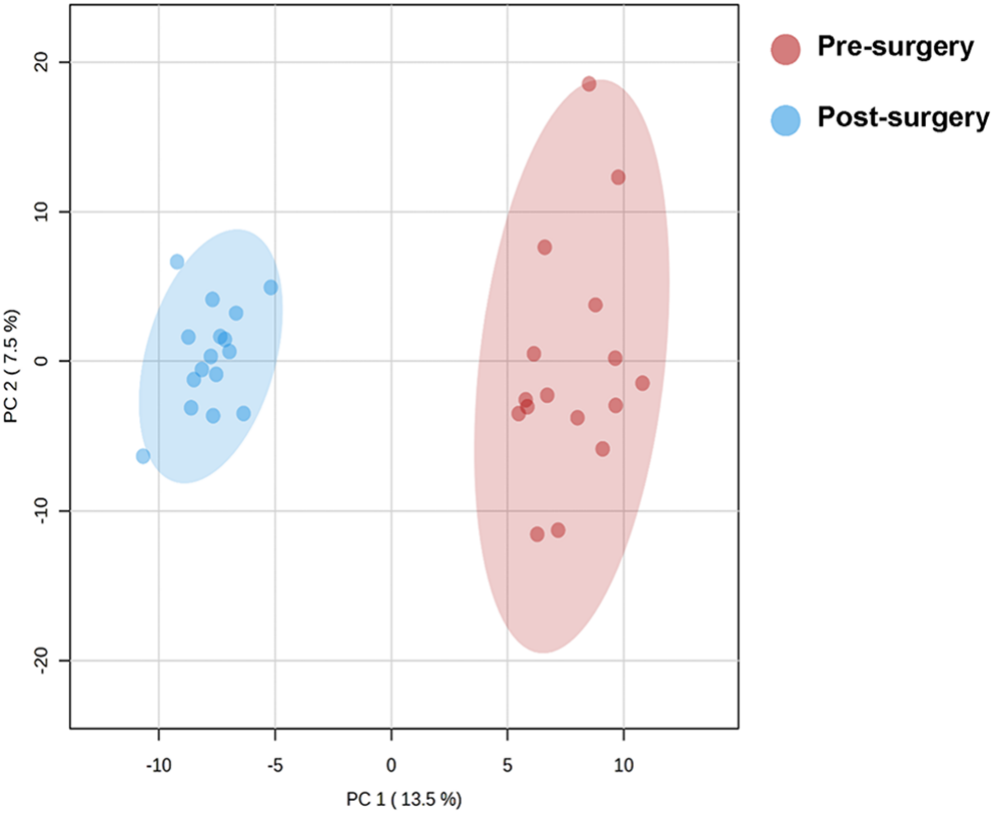
Principal Component Analysis (PCA) illustrating the distinction between prebariatric surgery (red) and postbariatric surgery (blue) urine proteomic profiles. Each dot represents an individual sample, with 95% confidence ellipses demonstrating the separation between groups. The analysis reveals a significant shift in protein composition following bariatric surgery, as confirmed by PERMANOVA (*F* = 49.54, R^2^ = 0.62283, *p* = 0.001).

### Analysis of the Most Altered Proteins

Among the 1016 proteins found to be altered after surgery, 42 were increased, while 12 proteins were decreased by at least 50% (≥1.5-fold change, Figure 3). A heatmap analysis of these altered proteins is shown in Figure 4. Proteins were then grouped according to their functional category, ordered from lowest to highest *p*-value, and divided according to whether their relative abundance is increased or decreased 2 years after surgery (Table 2). Functional classification of the top most altered proteins revealed that these were predominantly associated with immune function (*n* = 16; e.g., protein S100-A9, α-1-acid glycoproteins 1 and 2); cytoskeleton/cell adhesion (*n* = 12; e.g., supervillin, ezrin, periplakin); kidney adaptation (*n* = 11; e.g., elongation factor 1-α 1, cystatin-B, megalin, cubilin); lipid transport (*n* = 7; e.g., apolipoproteins E and A-IV), besides proteins of diverse functional classes, including hormone binding, micronutrient transport, and glucose metabolism (*n* = 18).

**Figure 3:**
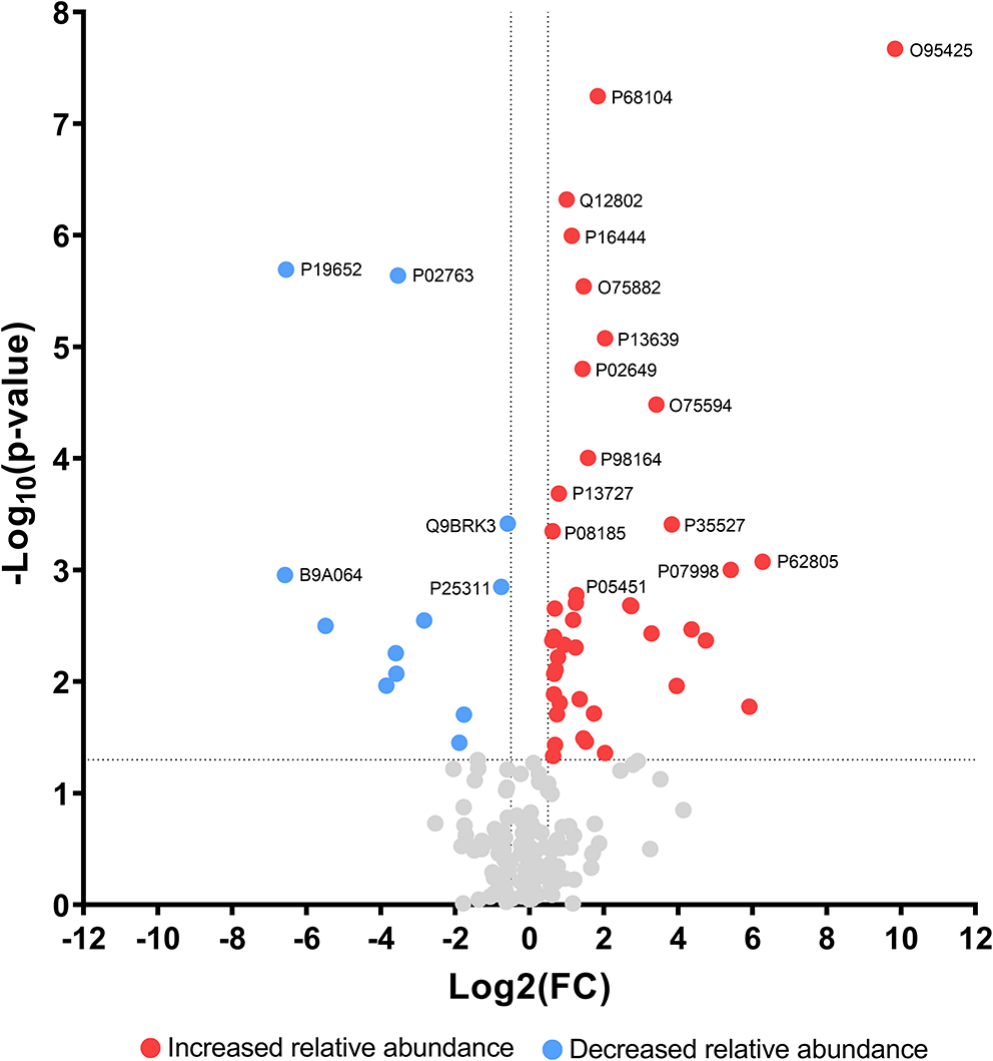
Volcano plot of urinary protein relative abundance pre- and postbariatric surgery. The *x*-axis represents the log_2_ fold-change (FC) in protein relative abundance, with positive values indicating higher abundance postsurgery and negative values indicating lower abundance postsurgery. The *y*-axis represents the -log_10_(*p*-value), where higher values correspond to greater statistical significance (lowest *p-*value). Proteins with at least 50% increase in relative abundance postsurgery are highlighted in red (≥1.5 FC), while those with at least 50% decrease in relative abundance are represented in blue (≤−1.5 FC). The top 20 most altered proteins are labeled with their respective Accession IDs (UniProtKB). Proteins in gray are not significantly changed.

**Figure 4:**
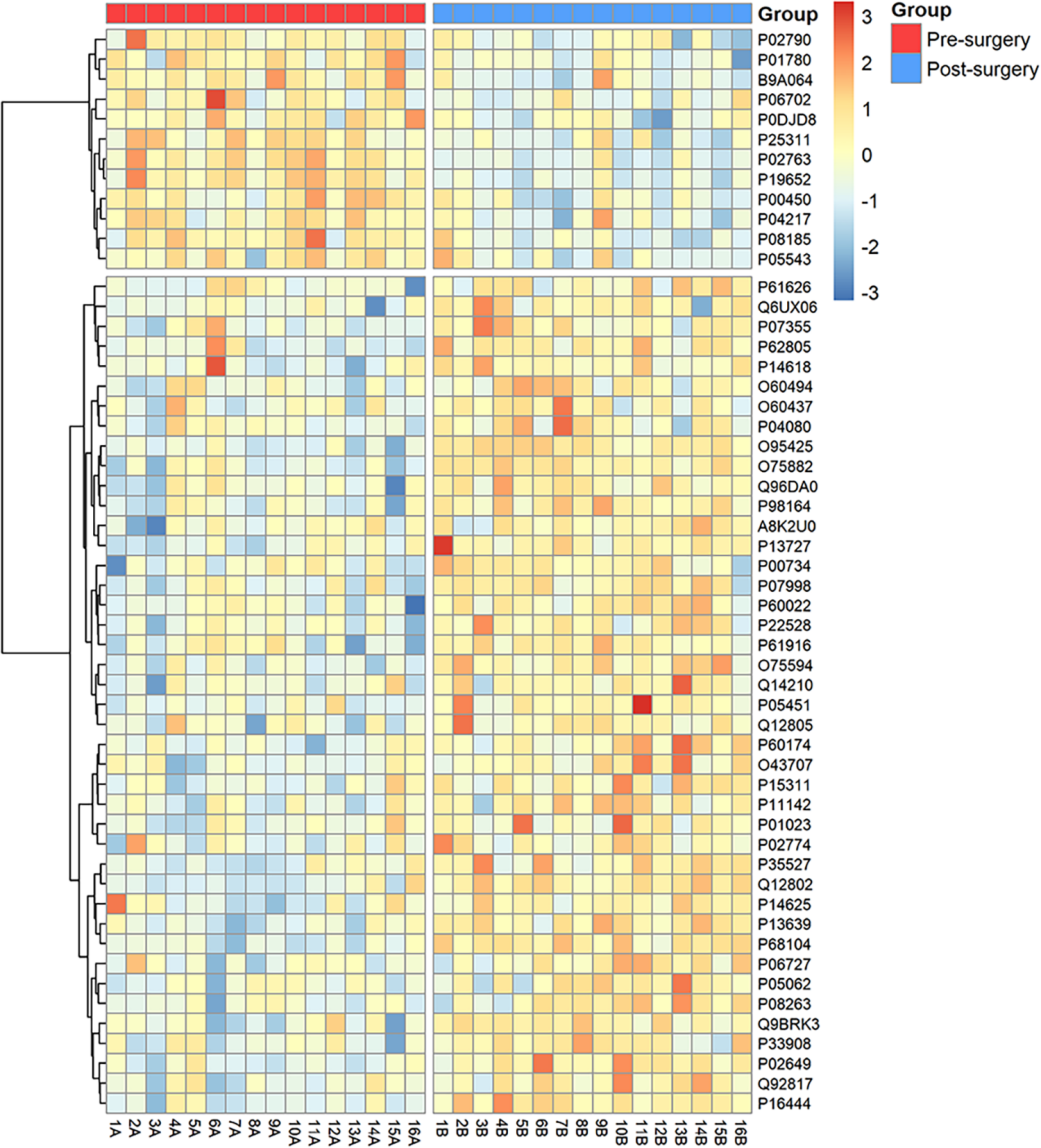
Heatmap analysis of the top 54 most altered urinary proteins (≥1.5-fold-change and *p* < 0.05) between pre- and postbariatric surgery. Each row represents an individual protein codified by the Accession ID (UniProtKB), while numbered columns correspond to each patient at pre- (A) and postsurgery (B). The color gradient indicates relative abundance (red = higher, blue = lower).

**Table 2:** Urinary Proteins Exhibiting Significantly Altered Abundance after Weight Loss Induced by Bariatric Surgery, Organized into Functional Categories and Ordered by Lowest p-value^a^

**Immune Response**			**Cytoskeleton/Cell Adhesion**			**Kidney Adaptation**		
**ID**	**Name**	**-Log_10_(p-value)**	**ID**	**Name**	**-Log_10_(p-value)**	**ID**	**Name**	**-Log_10_(p-value)**
**Increased abundance postsurgery**								
P16444^b^	Dipeptidase 1	5.9973	O95425	Supervillin	7.6724	P68104	Elongation factor 1-α 1	7.2471
O75882^b^	Attractin	5.5437	P35527	Keratin, type I cytoskeletal 9	3.4089	P16444^b^	Dipeptidase 1	5.9973
O75594	Peptidoglycan recognition protein 1	4.4835	Q9BRK3^b^	Matrix remodeling-associated protein 8	3.3487	O75882^b^	Attractin	5.5437
P13727	Bone marrow proteoglycan	3.6864	Q96DA0	Zymogen granule protein 16B	2.6768	P13639	Elongation factor 2	5.0775
P60022	β-defensin 1	2.6853	P15311	Ezrin	2.4687	P98164^b^	Megalin	4.0067
P08263^b^	Glutathione S-transferase A1	2.,6553	P61916	NPC intracellular cholesterol transporter 2	2.4044	Q9BRK3^b^	Matrix remodeling-associated protein 8	3.3487
P11142	Heat shock cognate 71 kDa protein	2.4332	Q92817	Envoplakin	2.3707	Q6UX06^b^	Olfactomedin-4	2.2182
Q14210	Lymphocyte antigen 6D	2.0707	O43707	α-actinin-4	2.3298	O60494^b^	Cubilin	1.8095
P61626	Lysozyme C	1.4916	P07355^b^	Annexin A2	2.3066	P04080	Cystatin-B	1.4332
P01023	α-2-macroglobulin	1.463	Q6UX06^b^	Olfactomedin-4	2.2182			
			Q12805	EGF-containing fibulin-like extracellular matrix protein 1	1.8877			
			O60437	Periplakin	1.3608			
**Decreased abundance postsurgery**								
P19652^b^	α-1-acid glycoprotein 2	5.6926				P19652^b^	α-1-acid glycoprotein 2	5.6926
P02763^b^	α-1-acid glycoprotein 1	5.6411				P02763^b^	α-1-acid glycoprotein 1	5.6411
P04217	α-1B-glycoprotein	2.5013						
B9A064	Immunoglobulin lambda-like polypeptide 5	2.9574						
P01780	Immunoglobulin heavy variable 3–7	1.9654						
P06702	Protein S100-A9	1.7048						

**Lipid Metabolism**			**Other**		
**ID**	**Name**	**-Log_10_(p-value)**	**ID**	**Name**	**-Log_10_(p-value)**
**Increased abundance postsurgery**					
P02649	Apolipoprotein E	4.8041	Q12802	A-kinase anchor protein 13	6.3206
P98164^b^	Megalin	4.0067	P62805	Histone H4	3.074
P08263^b^	Glutathione S-transferase A1	2.6553	P07998	Ribonuclease pancreatic	3.0018
P07355^b^	Annexin A2	2.3066	P05451	Lithostathine-1-α	2.7768
P06727	Apolipoprotein A-IV	1.843	P60174	Triosephosphate isomerase	2.7067
O60494^b^	Cubilin	1.8095	P22528	Cornifin-B	2.5531
			P33908	Mannosyl-oligosaccharide 1,2-α-mannosidase IA	2.3704
			P05062	Fructose-bisphosphate aldolase B	2.1084
			P00734	Prothrombin	1.9631
			P14618	Pyruvate kinase PKM	1.7754
			A8K2U0	α-2-macroglobulin-like protein 1	1.7143
			P14625	Endoplasmin	1.708
			P02774	Vitamin D-binding protein	1.3357
**Decreased abundance postsurgery**					
P25311	Zinc-α-2-glycoprotein	2.8507	P08185	Corticosteroid-binding globulin	3.417
			P00450	Ceruloplasmin	2.5495
			P02790	Hemopexin	2.2549
			P0DJD8	Pepsin A-3	2.0711
			P05543	Thyroxine-binding globulin	1.4543

a Each entry includes the UniProt identifier (ID) and the corresponding protein name.

b Present in more than one functional category.

## Discussion

Weight loss achieved through BS is associated with significant metabolic changes, but how these processes influence the urinary proteome has remained unexplored. Herein, we performed untargeted LC-MS-based urinary proteomics in 16 patients with obesity before and 2 years after BS in order to identify systemic modifications and potentially nephroprotective effects induced by weight loss. To minimize confounding factors, we excluded patients with diabetes, chronic inflammatory diseases, or overt kidney diseases, thereby reducing the likelihood of urinary proteomic changes influenced by factors beyond obesity.

Using this approach, we were able to identify a significantly altered abundance in more than 1000 proteins after weight loss. A clear separation between the proteomics profile of the same individual pre- and postsurgery was observed, suggesting an extensive shift in urinary protein composition following substantial weight loss. From these, the 54 proteins with at least a 50% fold-change alteration in relative abundance postsurgery, highlighted in Figure 3, deserved special consideration in our analysis. From those, the majority were related to the immune system. Obesity is recognized for its association with a chronic low-grade inflammatory state and immune system dysfunction.^32^ There is a large volume of accumulated evidence that weight loss following BS leads to adaptations of immune cell populations and allows improvements in inflammatory markers.^21,22^ In our analysis, we found that the relative abundance of proteins such as dipeptidase 1, attractin, and peptidoglycan recognition protein 1 had increased 2 years postsurgery. Dipeptidase 1 has a role in immune cell migration;^33^ attractin regulates the activity of chemokines^34^ and peptidoglycan recognition protein 1 is an innate immunity protein that modulates acquired immunity and plays a crucial role in the recognition of the bacterial peptidoglycan and antimicrobial defense.^35^ Conversely, the decrease in acute-phase proteins like α-1-acid glycoproteins (α1-AGP) 1 and 2 points to a reduction of the systemic inflammatory state that characterizes obesity.^36^ Therefore, our study findings further support the previously reported decrease of systemic inflammation following significant weight loss induced by BS.^37^ Moreover, proteins involved in immune response associated with unfavorable outcomes in the context of kidney diseases were also found to be less abundant, namely immunoglobulin heavy variable 3–7 (IGHV3–7), which has been linked to immune response in kidney transplantation,^38^ and immunoglobulin lambda-like polypeptide 5 (IGLL5), which has been shown to be increased in the setting of clear renal cell carcinoma.^39^

Inflammatory states are known to generate oxidative stress, while heat shock proteins play a paramount role in protecting cells from reactive oxygen species.^40,41^ In our study, the relative abundance of heat shock cognate 71 kDa protein (HSPA8) increased, while protein S100-A9 was found to be less abundant 2 years postsurgery. S100 protein levels are increased in response to oxidative stress^42,43^ and were demonstrated to correlate with metabolic risk score, adipocyte size, and insulin resistance in obesity.^44^ Furthermore, the urinary heterodimer formed by S100-A8 and S100-A9, calprotectin, has been associated with acute kidney injury structural damage,^45^ tubular epithelial cell injury, and inflammation.^46^ Overall, our results suggest that obesity-induced inflammation and oxidative stress are ameliorated following BS-induced weight loss.

A considerable number of urinary proteins found to be highly altered after weight loss are associated with the cytoskeleton and cell adhesion. These included the increased abundance of supervilin, a protein that forms a high-affinity link between the actin cytoskeleton and the cellular membranes and is expressed by a variety of cells and tissues;^47^ ezrin and keratin type I cytoskeletal 9 (K1C9) that integrate cytoskeleton connections;^48,49^ annexin A2, a protein involved in the formation of tight junctions; envoplakin and peroplakin that are involved in the link between desmosomes and intermediate filaments;^50,51^ NPC intracellular cholesterol transporter 2 (NPC2) that plays a critical role in intracellular cholesterol transport and homeostasis, and is also involved in maintaining cytoskeletal integrity, particularly through its regulatory function in membrane repair.^52^ Interestingly, some of the proteins that participate in the cytoskeleton and cell adhesion, identified to increase after weight loss, were also demonstrated to have a crucial role in maintaining the integrity of the kidney filtration barrier. Among those are annexin A2, a protein that participates in podocyte cytoskeletal rearrangement and whose altered levels had been reported in several kidney diseases associated with proteinuria,^53,54^ and the actin binding protein α-actinin-4 (ACTN4), which is predominantly expressed in podocytes where it plays a role in maintaining the glomerular structural integrity.^55^ In fact, mutations in the *ACTN4* gene have been linked to familial forms of focal segmental glomerulosclerosis, which is characterized by proteinuria and progressive kidney dysfunction.^56^ EGF-containing fibulin-like extracellular matrix protein 1 (EFEMP1), an extracellular matrix glycoprotein with a protective role against oxidative stress, had also been implicated in preventing fibrotic processes in several organs, including the kidney,^57^ while Zymogen granule protein 16B (ZG16B) was shown to have endothelial activity, promoting vascular permeability and angiogenesis.^58^ Additionally, matrix remodelling-associated protein 8 (MXRA8), a protein involved in extracellular matrix dynamics and cell adhesion identified in the kidney tissue, was also increased, thereby possibly intervening in maintaining renal structural integrity.^59^ Taken together, our findings suggest that the urinary proteome changes linked to the cytoskeleton and cell adhesion may reflect protective changes in the kidney from obesity-related kidney disease. Indeed, obesity-related glomerulopathy is closely connected to cytoskeleton changes in glomerular cells, such as the podocytes, which may culminate in loss of glomerular adhesion,^60^ and significantly affect the glomerular slit diaphragm integrity.^61,62^ As a consequence of obesity, these changes may be driven by factors such as hyperfiltration, inflammatory changes, and ectopic lipid accumulation.^3^

Several urinary proteomic changes identified in this study may also reflect kidney functional adaptations. One example is dipeptidase 1, which within the kidney participates in the metabolism of glutathione and its conjugates,^63^ while contributing to the conversion of leukotriene D4 (LTD4) into leukotriene E4 (LTE4)^64^ and thereby attenuating inflammatory responses that affect the renal smooth muscle vascular tone.^65^ Indeed, cysteinyl leukotrienes, such as LTD4, reduce renal blood flow and the glomerular filtration rate by triggering vasoconstriction.^66^ Attractin, which was found to be increased 2 years postsurgery, could also be involved in the mechanisms leading to weight loss-related kidney protection. As a demonstration of the paramount role of this protein for the kidney structure, attractin-deficient mice have a severe loss of extracellular proteoglycans between kidney tubules in addition to a loss of glycosylated material within the intratubular brush border.^67^ The reduction in the abundance of α-1-acid glycoproteins (α1-AGP) 1 and 2, which are known to affect vascular permeability, unveils another potential mechanism for the nephroprotective effects of weight loss in patients with obesity. In a cross-sectional study with 2579 female patients, serum α1-AGP1 was significantly and positively associated with urinary albumin-to-creatinine ratio,^68^ which, when increased, is an early sign of kidney dysfunction. In children with obesity, a strong correlation between α1-AGP1 and urinary albumin-to-creatinine ratio was also found, suggesting that high levels of α1-AGP1 before the onset of albuminuria may be useful as a biomarker of early glomerular damage.^69^ Bone marrow proteoglycan abundance was also found to increase 2 years postsurgery. Extracellular matrix proteoglycans are responsible for tissue organization, stability, and differentiation.^70^ The increased urinary abundance of bone marrow proteoglycan 2 years postsurgery suggests the occurrence of tissue remodelling. Although bone marrow proteoglycan has not been shown to be directly involved in these phenomena in the kidney, nor proved otherwise, it has been identified in the kidney tissue,^71^ while there is no doubt that there is an important interplay between bone marrow activity and kidney function. In our patient cohort, creatinine clearance was above the upper reference limit, suggesting a tendency toward hyperfiltration, a common finding in obesity.^72^ In contrast, serum creatinine, urea, urinary albumin, and protein excretion remained within the reference ranges, indicating no overt impairment of kidney function. Since obesity is known to induce renal injury at the cellular level well before overt clinical manifestations emerge,^5^ the observed protein alterations may represent part of the molecular substrate underlying these silent, preclinical disease processes, warranting further validation.

Low-density lipoprotein receptor-related protein 2, also known as megalin, and cubilin were found to be more abundant postsurgery. Megalin acts together with cubilin to mediate the transport of lipoprotein transport. Both proteins are present in the kidney proximal tubules, mediating the transport of several substances, including lipids.^73^ These proteins also play a crucial role in the reabsorption of albumin and low-molecular-weight proteins filtered through the glomerulus.^74^ Notably, reduced levels of these receptors have been reported in conditions such as kidney ischemia-reperfusion injury^75^ or in chronic kidney disease,^76^ contributing to elevated urinary albumin levels in proteinuric kidney diseases. Modulation of megalin levels appears to be part of the adaptive response of tubular cells aimed at cytoprotection and regeneration, which led to the proposal of being used as a potential therapeutic target for the treatment of kidney disorders associated with proteinuria.^74^ In the context of weight loss after BS, the increased abundance of these proteins, alongside the expected reduction in albuminuria,^77^ suggests their putative enrolment in the restoration of the kidney functional integrity. The elongation factors 1-α 1 (EF1A) and 2 (EF2), proteins involved in translation, elongation, and cytoskeleton regulation, are shown to be increased after weight loss, also suggesting the enactment of nephroprotective mechanisms, since EF1A and EF2 are diminished in senescent proximal tubular epithelial cells and apoptosis.^78^

Obesity is known to have profound effects on multiple endocrine and metabolic functions, which to a large extent can be restored by weight loss.^79^ Besides the proteins mentioned above, the relative abundance of several other proteins that participate in systemic processes was found to be altered after weight loss. Among these proteins, there are quite a few involved in modulating lipid metabolism, endocrine function, and micronutrient transport, possibly reflecting systemic adaptations. Of particular notice are the effects on lipid metabolism that also became evident in the urinary proteomic profile. Weight loss after BS was associated with an improvement of the atherogenic lipid profile, with a significant decrease of LDL Cholesterol and a clinically relevant increase of HDL Cholesterol. Apolipoprotein E (APOE), which is involved in the transport of triglyceride-enriched lipid particles between organs, and Apolipoprotein A-IV, a major component of HDL cholesterol, were found to be more abundant postsurgery, further reflecting the improvement of the atherogenic lipid profile of these patients.^80^

To the best of our knowledge, this study is the first to provide a detailed urinary proteomic profile of changes following weight loss induced by BS. While long-term durability of weight loss and effective remission and prevention of several major obesity-related disorders after BS have been extensively explored by previous studies,^81^ our findings on urine proteomics provide data toward a deeper understanding of the systemic adaptations underlying weight loss and enabled the identification of proteins associated with obesity and weight loss, which could be involved in kidney lesion or protection, respectively.

Our study also has limitations to be acknowledged. This study included a small number of participants submitted to different BS procedures, which may limit the generalizability of the findings and preclude any further subgroup analysis. However, a power calculation based on a paired design (two-tailed paired *t*-test), with power = 0.80, α = 0.05, and an effect size threshold for large effects (*d* = 0.8), indicates that a sample size of *n* = 16 achieves sufficient power (≈85%) to detect such differences. Importantly, because our design was paired — with each participant serving as their own control — the intraindividual comparison reduces interindividual variability and thereby increases statistical power relative to an independent group design. We therefore consider that the sample size employed in this study is adequate and provides robust evidence supporting the relevance of our findings. Moreover, patients with relatively common comorbidities associated with obesity, such as diabetes, osteoarthritis, and obesity-related kidney dysfunction, were excluded from the study; to what extent the concomitant presence of these conditions influences the urinary proteome remains to be disclosed and will require dedicated research efforts in the future. BS alters both macro- and micronutrient intake and absorption. In addition to producing significant reductions in body fat percentage, it also impacts other components of body composition, including muscle mass.^82,83^ This may help explain the statistically significant mean variation of 0.3 g/dL observed between pre- and postsurgery total serum protein levels, although values remained within the reference range. Additionally, as our cohort was predominantly female, subgroup analyses are underpowered to identify any sex-specific changes. As male and female urinary proteome profiles are known to differ, future studies with larger and more balanced cohorts are warranted to explore potential sex-specific adaptations. Lastly, although several proteins putatively involved in neproprotective processes were identified, further validation is still necessary to confirm the roles of the identified proteins in this specific disease model. However, key strengths should be noted. Namely, the fact that patients were used as their own controls, minimizing individual variability, patients with other medical conditions that could introduce major bias were excluded, and the use of high-resolution proteomic analysis enabled sensitive protein detection.

In summary, our study provides a comprehensive urinary proteomic profile of patients with obesity before and 2 years after weight loss induced by BS. Our findings reveal that substantial systemic and kidney adaptations occurring after significant weight loss following BS are reflected in changes in the urinary proteome. The most prominent changes involved proteins related to immune system regulation, oxidative stress, cytoskeletal organization, and kidney-specific functions. We observed a decrease in inflammation markers alongside an increase in proteins associated with immune modulation and oxidative stress protection. Additionally, we found changes in cytoskeletal proteins and extracellular matrix components related to kidney function, suggesting tissue remodeling and its potential involvement in nephroprotective mechanisms.

## Supplementary Material





## Data Availability

The mass spectrometry proteomics data have been deposited to the ProteomeXchange Consortium via the PRIDE^29^ partner repository with the data set identifier PXD063904 and 10.6019/PXD063904. Supporting Tables containing the full list of identified, quantified, and significantly altered proteins are provided as part of the Supporting Information.

## Notes

Identified and quantified urinary proteins pre- and postbariatric surgery (Table S1); statistically analyzed urinary proteins with relative abundance data (Table S2); differentially abundant urinary proteins after bariatric surgery (≥1.5-Fold-Change, *p* < 0.05) (Table S3) (XLSX)
Principal Component Analysis (PCA) illustrating the distinction between prebariatric surgery and postbariatric surgery urine proteomic profiles stratified by sex (Figure S1) (PDF)

## Abbreviations

ACEi: Angiotensin-Converting Enzyme Inhibitors
ACTN4: α-Actinin-4
APOE: Apolipoprotein E
ARBs: Angiotensin II Receptor Blockers
BMI: Body Mass Index
BS: Bariatric Surgery
CKD-EPI: Chronic Kidney Disease Epidemiology Collaboration
EF1A: Elongation Factor 1-α 1
EF2: Elongation Factor 2
EFEMP1: EGF-Containing Fibulin-Like Extracellular Matrix Protein 1
eGFR: Estimated Glomerular Filtration Rate
GFR: Glomerular Filtration Rate
HbA1c: Glycated Hemoglobin
HDL: High-Density Lipoprotein
LC-MS: Liquid Chromatography–Mass Spectrometry
LFQ: Label-Free Quantification
LDL: Low-Density Lipoprotein
LoDs: Limits of Detection
LTD4: Leukotriene D4
LTE4: Leukotriene E4
MS: Mass Spectrometry
MXRA8: Matrix Remodeling-Associated Protein 8
NPC2: NPC Intracellular Cholesterol Transporter 2
PCA: Principal Component Analysis
PERMANOVA: Permutational Multivariate Analysis of Variance
RYGB: Roux-en-Y Gastric Bypass
SADIS-S: Single Anastomosis Duodenal-Ileal Bypass with Sleeve Gastrectomy
α1-AGP: α-1-Acid Glycoprotein
%EBMIL: Percentage of Excess BMI Loss
%TWL: Percentage of Total Weight Loss
