# Supplementary material for: Untargeted Urinary Proteomics Uncovers Nephroprotective and Systemic Adaptations after Obesity Surgery-Induced Weight Loss
Source: J Proteome Res. 2025 Dec 10;25(1):119–30. doi: 10.1021/acs.jproteome.5c00500 (PMC12818830; doi:10.1021/acs.jproteome.5c00500)
Supplement: Supplementary file 2 [file pr5c00500_si_002.pdf]

Untargeted Urinary Proteomics Uncovers Nephroprotective and Systemic Adaptations after Obesity Surgery Induced Weight Loss

Pedro R. Pereira[1,2,3,*], David F. Carrageta[2,3,4], Bárbara Guerra-Carvalho[2,3,5], Patrícia C. Braga[2,3], João Pereira[2,3], Sofia S. Pereira [2,3], Mário Nora[6], Marta Guimarães[2,3,6], Anabela Rodrigues[1,2,3], Mariana P. Monteiro [2,3]

[1]Department of Nephrology, Unidade Local de Saúde de Santo António (ULS Santo António) - Porto, Portugal. (Largo Professor Abel Salazar, 4099-001 Porto, Portugal)

[2]Unit for Multidisciplinary Research in Biomedicine (UMIB), School of Medicine and Biomedical Sciences (ICBAS), University of Porto, Porto, Portugal. (Rua de Jorge Viterbo Ferreira n.º 228. 4050-313, Porto, Portugal)

[3] ITR - Laboratory for Integrative and Translational Research in Population Health, Porto, Portugal (Rua das Taipas 135, 4050-600, Porto, Portugal)

[4] Polytechnic Institute of Portalegre, Portalegre, Portugal (Praça do Município 11, 7300-110 Portalegre, Portugal)

[5] LAQV-REQUIMTE, Department of Chemistry, University of Aveiro, Aveiro, Portugal, (Campus Universitário de Santiago, 3810-193 Aveiro, Portugal)

[6] General Surgery Department and CRI for the surgical Treatment of Obesity and Metabolic Diseases, ULS Entre o Douro e Vouga, Santa Maria da Feira, Portugal (Rua Dr. Cândido de Pinho, 4520-221, Santa Maria da Feira, Aveiro)

***Corresponding author:**

Pedro R. Pereira - Department of Nephrology, Largo Professor Abel Salazar, 4099-001 Porto, Portugal, pedroreisper@gmail.com

Table of Contents

Supplementary Table 1 – Identified and Quantified Urinary Proteins Pre- and Post-Bariatric Surgery (XLSX)

Supplementary Table 2 – Statistically Analyzed Urinary Proteins with Relative Abundance Data (XLSX)

Supplementary Table 3 – Differentially Abundant Urinary Proteins After Bariatric Surgery (≥1.5 Fold Change, p<0.05) (XLSX)

Figure S1. Principal Component Analysis (PCA) illustrating the distinction between pre-bariatric surgery and post-bariatric surgery urine proteomic profiles stratified by sex.

Supplementary Figure 1 (S1)

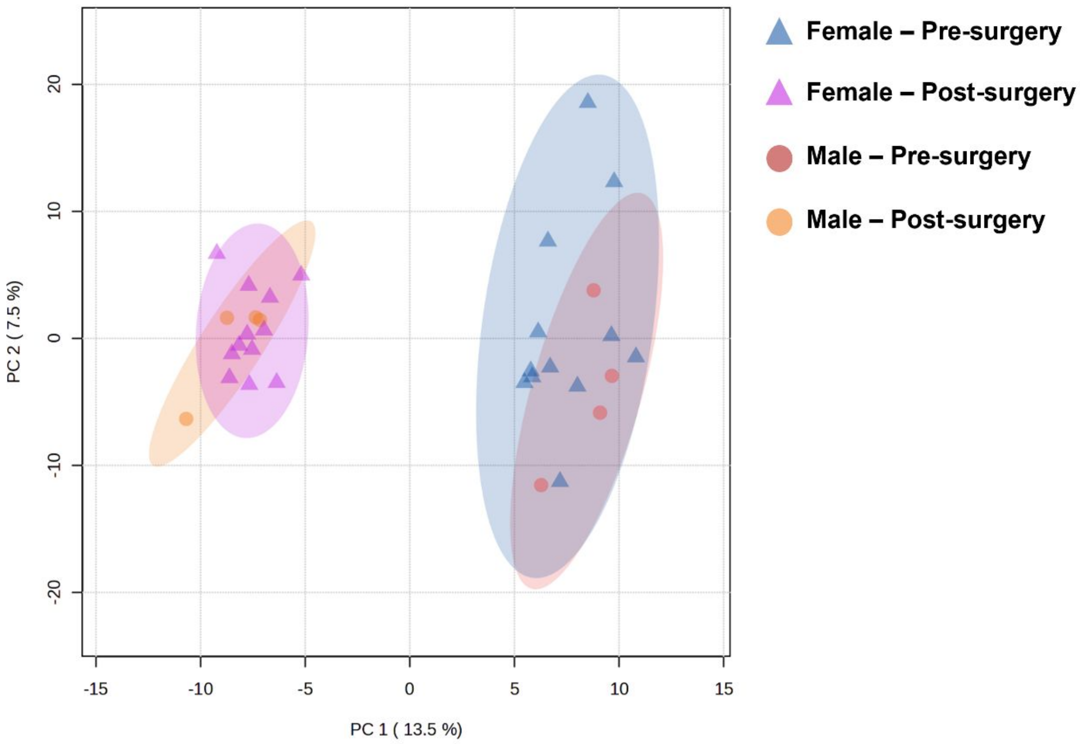

Figure S1 – Principal Component Analysis (PCA) illustrating the distinction between pre-bariatric surgery and post-bariatric surgery urine proteomic profiles stratified by sex. Female participants (n = 12) urine proteomics profile pre-bariatric surgery is represented by blue triangles while post-bariatric surgery is represented by pink triangles. Male participants (n = 4) urine proteomics profile pre-bariatric surgery is represented by red dots while post-bariatric surgery is represented by yellow dots. Each symbol (dot or triangle) represents an individual sample. A complete overlap of the cluster's 95% confidence ellipses corresponding to female and male participants is observed either pre-bariatric surgery or post-bariatric surgery. The analysis reveals a significant shift in protein composition following bariatric surgery despite stratification by sex, as confirmed by PERMANOVA ($F = 49.54$, $R^2 = 0.62283$, $p = 0.001$).
